# Differential Effectiveness of Hypothermic Targeted Temperature Management According to the Severity of Post-Cardiac Arrest Syndrome

**DOI:** 10.3390/jcm10235643

**Published:** 2021-11-30

**Authors:** Kazuya Kikutani, Mitsuaki Nishikimi, Tatsutoshi Shimatani, Michihito Kyo, Shinichiro Ohshimo, Nobuaki Shime

**Affiliations:** Department of Emergency and Critical Care Medicine, Graduate School of Biomedical and Health Sciences, Hiroshima University, Hiroshima 734-8551, Japan; kikutani@hiroshima-u.ac.jp (K.K.); tatsutoc@gmail.com (T.S.); mkyo@hiroshima-u.ac.jp (M.K.); ohshimos@hiroshima-u.ac.jp (S.O.); nshime@hiroshima-u.ac.jp (N.S.)

**Keywords:** post-cardiac arrest syndrome, targeted temperature management, therapeutic hypothermia, cardiopulmonary resuscitation, risk classification

## Abstract

International guidelines recommend targeted temperature management (TTM) to improve the neurological outcomes in adult patients with post-cardiac arrest syndrome (PCAS). However, it still remains unclear if the lower temperature setting (hypothermic TTM) or higher temperature setting (normothermic TTM) is superior for TTM. According to the most recent large randomized controlled trial (RCT), hypothermic TTM was not found to be associated with superior neurological outcomes than normothermic TTM in PCAS patients. Even though this represents high-quality evidence obtained from a well-designed large RCT, we believe that we still need to continue investigating the potential benefits of hypothermic TTM. In fact, several studies have indicated that the beneficial effect of hypothermic TTM differs according to the severity of PCAS, suggesting that there may be a subgroup of PCAS patients that is especially likely to benefit from hypothermic TTM. Herein, we summarize the results of major RCTs conducted to evaluate the beneficial effects of hypothermic TTM, review the recent literature suggesting the possibility that the therapeutic effect of hypothermic TTM differs according to the severity of PCAS, and discuss the potential of individualized TTM.

## 1. History of RCT for the Setting Temperature during TTM

Fever after cardiac arrest and resuscitation is common [1] and can exacerbate brain damage in patients with post-cardiac arrest syndrome (PCAS), which is known as a severe syndrome caused by systemic ischemia–reperfusion after cardiac arrest resuscitation [2]. A few studies have shown that the occurrence of fever in post-cardiac arrest patients was associated with increased mortality [1] and poor neurological prognosis [3].

Hypothermic targeted temperature management (TTM) in adult post-cardiac arrest patients has been widely used in clinical practice, ever since the results of two trials published in 2002 suggested its therapeutic benefit [4,5]. In 2013, Nielsen et al. reported, on the basis of the results of a large randomized controlled trial (RCT) (TTM-1 trial), that there was no difference in the survival or likelihood of a favorable neurological outcome between TTM at 33 °C and TTM at 36 °C in post-cardiac arrest patients with presumed cardiogenic cardiac arrest [6]. The TTM-1 trial differed from the previous aforementioned RCTs in that measures were taken to control fever and to maintain normothermia (normothermic TTM), while in the previous two RCTs, no measures were taken for fever control. Considering the results of the TTM-1 trial, current guidelines recommend that the temperature setting for TTM should be in the range of 32 °C to 36 °C, with the actual temperature setting left to the preference of the treating physicians [7]. Therefore, many hospitals changed their protocols from hypothermic TTM to normothermic TTM, because normothermic TTM is generally less invasive and is associated with fewer complications. However, a few subsequent studies have reported interesting results in that the prognosis of the patients became worse in some of the hospitals that changed their protocol from hypothermic to normothermic TTM [8,9]. Thus, the optimal temperature setting temperature for TTM—hypothermic or normothermic—still remains unclear.

Two well-designed RCTs were conducted recently in an attempt to resolve the question. In the first RCT conducted in 2019 in 25 ICUs in France (HYPERION trial), the effects of TTM at 33 °C and 37 °C were compared in post-cardiac arrest patients with a non-shockable rhythm. In this trial, a higher survival rate and a higher rate of a favorable neurological outcome at 90 days (defined as Cerebral Performance Category ≤2) were observed in the group that received hypothermic TTM at 33 °C for 24 h compared to the group that received normothermic TTM at 37 °C for 24 h [10]. The second trial was the more recent international trial conducted in 2021 in 61 institutes, mainly in Europe (TTM-2 trial), by the same research group that conducted the TTM-1 trial [11]. This study failed to show any benefit of hypothermic TTM, in terms of survival or the likelihood of a favorable neurological outcome, as compared to normothermic TTM. In addition, a recent RCT showed no difference in the beneficial effects between moderate hypothermia (TTM at 31 °C) and mild hypothermia (TTM at 34 °C) among hypothermic TTM [12].

## 2. Possible Reasons for the Discrepant Results among the RCTs

The discrepant results between the HYPERION and TTM-2 trials could possibly be explained by the difference in the distribution of the disease severity between the postcardiac arrest patient cohorts included in the two trials [10,11]. Approximately 60% of patients had circulatory failure in the HYPERION study as compared to 30% of patients in the TTM2 study. While the 3-month mortality rate in the HYPERION trial was over 80%, the 6-month mortality rate in the TTM-2 trial was only about 50%. In addition, only about 55% of the patients had a poor neurological prognosis at 6 months (defined with a modified Rankin scale score of ≥4) in the TTM-2 trial, while more than 90% had a poor neurological prognosis at 90 days (defined in a Cerebral Performance Category of ≥3) in the HYPERION trial. Thus, overall, the HYPERION trial included a larger number of patients with severe PCAS than the TTM-2 trial, which could explain the different results between the two trials. As the differential effect of some treatments by disease severity were reported in other clinical fields, the effectiveness of hypothermic TTM might also differ according to the severity of PCAS; we may be able to find a subgroup of PCAS patients for whom hypothermic TTM is especially beneficial if we can estimate their severity precisely.

## 3. Risk Classification for Estimating the PCAS Severity

**Figure 1:**
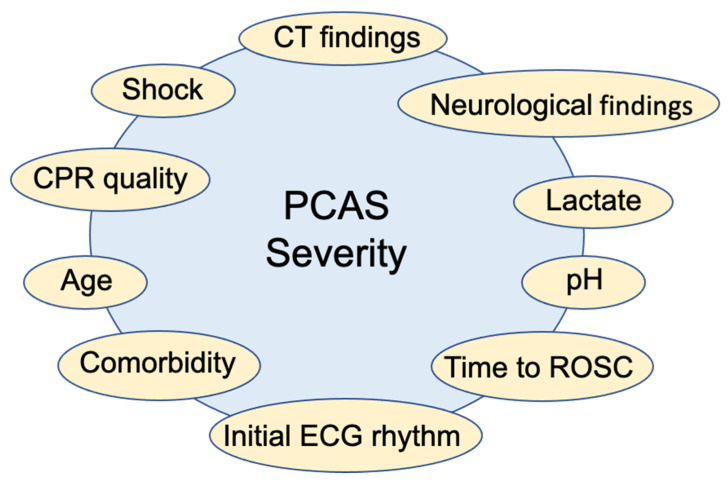
Factors affecting the severity of post-cardiac arrest syndrome.

Many of the studies that have been reported until now have estimated the severity of PCAS using a single clinical factor [13,14,15,16,17,18,19,20,21,22] (Figure 1), for example, time until ROSC [13,14,15], blood pH [16,17], lactate [16,18,19], and so forth. Brain computed tomography (CT) has been also used to predict the neurological prognosis in post-cardiac arrest patients [23,24]; the gray matter/white matter ratio (GWR) in brain CT has been shown to be a useful objective measure of PCAS severity [20,21,22]. However, the reported predictive accuracies of these single clinical factors from previous studies have been inconsistent, and these parameters cannot sufficiently discriminate between patients with a good prognosis and those with a poor prognosis. Thus, it was considered that a “suitable scaling method” based on a combination of prognostic factors would be useful [16,19,25,26,27].

Several predictive scores have been developed for a more precise estimation of the severity of PCAS [28,29,30,31,32,33,34,35,36] (Table 1). Coppler et al. published the Pittsburgh Cardiac Arrest Category (PCAC), a four-level illness severity score for PCAS composed of sub-parameters of the FOUR and SOFA scores [30,31]. Gue et al. proposed the NULL-PLEASE score, calculated from 10 clinical factors [32]. Kiehl EL et al. developed the C-GRApH based on the results of their multivariate analysis [33]. Our group previously proposed the post-Cardiac Arrest Syndrome for Therapeutic hypothermia score (CAST score), which is a predictive score focused on PCAS patients scheduled to undergo TTM [34,35]. The score consists of eight clinical variables, including the waveform on the initial electrocardiograph (ECG), time to return of spontaneous circulation (ROSC), score on the motor scale of the Glasgow coma scale (GCS), blood pH, serum levels of albumin, hemoglobin, and lactate, and the GWR on brain CT. Subsequently, considering the feasibility of use in actual clinical practice, we simplified the score to develop the revised CAST score (rCAST) as a risk classification tool for patients with PCAS scheduled to undergo TTM [36]. The rCAST score is calculated from five clinical factors prior to the initiation of TTM (initial ECG waveform, time to ROSC, score on the motor scale of the GCS, blood pH, and serum lactate). Our previous study showed that the risk stratification of patients on the basis of the rCAST score into low-, moderate-, and high-severity groups is useful for predicting the likelihood of a favorable neurological outcome in patients with PCAS scheduled to undergo TTM [36].

## 4. Differential Effectiveness of Hypothermic TTM According to the Severity of PCAS as Assessed by a Single Clinical Factor

The differential effect of hypothermic TTM according to the severity of PCAS as assessed by a single clinical factor has been well studied [37,38,39,40]. Kaneko et al. reported, based on a large database in Japan, that among patients who received hypothermic TTM, the neurological prognosis was better in patients with a time to ROSC of less than 30 min compared to those with a longer time to ROSC [37]. Another retrospective study reported that TTM at 32–34 °C improved the neurological outcome at 30 days compared to TTM at 35–36 °C in patients with severe hyperlactatemia (>12 mmol/L) and that the interaction for the outcome between the levels of lactate and the core temperature setting for TTM was significant [38]. Our retrospective study showed that PCAS patients who underwent hypothermic TTM had a better neurological outcome than PCAS patients who underwent normothermic TTM if they did not have any findings of hypoxic encephalopathy on brain CT [39].

In contrast, Kjaergaard et al. performed a secondary analysis of the data from the TTM-1 trial, which showed that TTM at 33 °C neither reduced the risk of mortality nor improved the neurological outcome as compared to TTM at 36 °C, regardless of the time to ROSC [40]. Therefore, as we have mentioned above, it may not be sufficient to consider any single clinical factor to determine the severity of PCAS, and risk classification based on a combination of clinical factors is needed for a more precise analysis of the differential effects of hypothermic TTM according to the severity of PCAS.

## 5. Differential Effects of Hypothermic TTM According to the Severity of PCAS as Assessed Using a Risk Score Based on Multiple Clinical Factors

To date, few studies have examined the differential effects of hypothermic TTM according to the severity of PCAS. Callaway et al. investigated whether the severity of PCAS—as determined using their risk classification system, PCAC—was associated with any change in the relationship between the target body temperature and patient prognosis [41]. In patients with mild to moderate coma (PCAC 1 or PCAC 2), TTM at 33 °C was associated with a lower survival rate than TTM at 36 °C; however, in patients with moderate coma and severe cardiopulmonary failure (PCAC 3) or severe coma (PCAC 4), TTM at 33 °C was associated with a better outcome than TTM at 36 °C [41]. In this analysis, the researchers excluded patients with severe cerebral edema or a highly malignant electroencephalogram, who are considered as belonging to the highest severity group in whom the outcomes seem to be poor, regardless of the TTM strategy used.

We evaluated the association between the core temperature setting for TTM and the neurologic outcomes in PCAS patients classified according to the rCAST score as having PCAS of high, moderate, and low severity, and examined the effect of TTM at 33–34 °C on the neurological outcome in each group [42]. Among the patients with PCAS of moderate severity, those undergoing TTM at 33–34 °C showed a better neurological prognosis than patients undergoing TTM at 35–36 °C, while no such difference in the prognosis according to the core temperature setting was observed in patients with mild or severe PCAS. These results suggest that the benefit of hypothermic TTM varies according to the severity of PCAS.

## 6. Who Are the Most Suitable Candidates for Hypothermic TTM?

**Figure 2:**
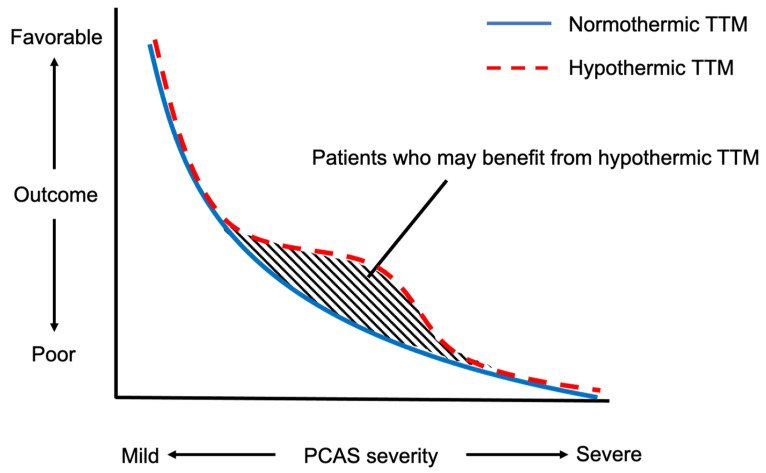
Hypothesis to explain the differential effectiveness of hypothermic target temperature management according to the severity of post-cardiac arrest syndrome.

Considering that previous animal studies indicated that hypothermic TTM provided no benefit if the brain ischemia had progressed beyond a certain level [43,44], PCAS patients with a poor prognosis are unlikely to show a favorable neurological outcome regardless of the treatment adopted, including hypothermic TTM. It may also not be surprising to consider that hypothermic TTM would be of no benefit to those with minimal or non-existent brain damage. Thus, the patient group that is especially likely to derive benefit from hypothermic TTM is the group with PCAS of moderate severity (Figure 2). This hypothesis is partly supported by the two recent clinical studies mentioned above [41,42], while additional RCT(s) would be needed to confirm this hypothesis.

Not only has the optimal temperature setting of TTM for each severity not been studied yet, but neither has the optimal time to reach the target body temperature, nor the optimal duration of the TTM for each severity, so future research addressing these issues is of great interest. Today, several new RCTs for TTM for post-cardiac arrest patients are ongoing, such as the ICECAP study, which is to compare the duration of hypothermic TTM [45]. These results are eagerly anticipated for a better understanding of the best strategy of TTM for post-cardiac arrest patients. Please also note that we focused on the beneficial effect of hypothermic TTM for patients with out-of-hospital cardiac arrest (OHCA) and did not mainly mention those with in-hospital cardiac arrest (IHCA). Because the characteristics of IHCA patients differ from OHCA patients [46,47], the evidence for hypothermic TTM in OHCA patients may not be directly applicable in IHCA patients. A large RCT to compare hypothermic TTM and normothermic TTM that focuses on the patients with IHCA is needed in the future.

## 7. Conclusions

The effect of hypothermic TTM may vary according to the severity of PCAS. The severity of PCAS should be evaluated by a combination of several factors, and several risk classifications are reported. A large RCT to compare the effect of hypothermic and normothermic TTM focusing on patients with PCAS of moderate severity is warranted.

## Figures and Tables

**Table 1:** Developed predictive scores and risk classifications for PCAS. Four score—full outline of unresponsiveness score; SOFA score—sequential organ failure assessment score; ESRD—end-stage renal disease; CAD—coronary artery disease; ROSC—return of spontaneous circulation; GCS—Glasgow coma scale; GWR—gray–white matter ratio.

Name	Reference	Variables	No of Variables	Simplified or Not (Do Not Need Electric Devices)	Risk Classification (More Than 3 Risk Groups)
OHCA score	[24]	Initial rhythm, no flow time, low flow time, lactate, creatinine	5	Not	Not shown
CAHP score	[25]	Age, location of cardiac arrest, initial rhythm, no flow time, low flow time, pH, epinephrin dose	7	Not	Low risk (score ≤ 150), Medium risk (score 150–200) High-risk group (score ≥ 200)
PCAC	[26,27]	Motor and brain stem scale of FOUR score, cardiovascular and respiratory scale of SOFA score	4	Simplified	Mild coma: PCAC 1 Moderate coma and mild cardiopulmonary failure: PCAC 2 Moderate coma and severe cardiopulmonary failure: PCAC 3 Severe coma: PCAC 4
NULL-PLEASE score	[28]	Initial rhythm, age, presence of witness, no flow time, low flow time, pH, lactate, past medical history of ESRD, still resuscitation, extra-cardiac cause	10	Simplified	Not shown
C-GRApH	[29]	Past medical history of CAD, glucose, initial rhythm, age, pH	5	Simplified	Low severity group: ≤1 Medium severity group: 2 and 3 High severity group: ≥4
CAST score	[30,31]	Initial rhythm, presence of witness and time to ROSC, motor scale of GCS, albumin, hemoglobin, pH, lactate, GWR	8	Not	Not shown
rCAST score	[32]	Initial rhythm, presence of witness and time to ROSC, motor scale of GCS, pH, lactate	5	Simplified	Low severity group: ≤5.5 Moderate severity group: between 6.0 and 14.0 High severity group: ≥14.5
